# Investigation into the metric properties of the workplace social capital questionnaire and its association with self-rated health and psychological distress amongst Greek-Cypriot registered nurses: cross-sectional descriptive study

**DOI:** 10.1186/s12889-018-5959-7

**Published:** 2018-08-23

**Authors:** Nicos Middleton, Panayiota Andreou, Maria Karanikola, Christiana Kouta, Ourania Kolokotroni, Evridiki Papastavrou

**Affiliations:** 1 Department of Nursing, School of Health Sciences, Cyprus University of Technology, 15 Vragadinou Str, 3041 Limassol, Cyprus; 2 St George University of London Medical School at the University of Nicosia, Nicosia, Cyprus

**Keywords:** Social capital, Workplace, Measurement, Construct validity, Psychological distress, Self-rated health

## Abstract

**Background:**

Social capital can been described as an individual or a collective attribute, with structural and cognitive components, and a bonding, bridging and linking typology. While extensively studied in the community, studies in occupational settings are sparse by comparison. Furthermore, there is no uniformity in its measurement. This study investigated the construct validity of a Workplace Social Capital questionnaire (WSC), originally developed in the Finnish Public Sector occupational cohort, in a different socio-cultural setting (Cyprus), language (Greek) and occupational group (Registered Nurses). It also explored its criterion concurrent validity according to observed association with self-rated health and psychological distress.

**Methods:**

Participants were 10% of all registered nurses (*N* = 362) who responded to the 8-item WSC scale during a nationwide educational programme. A unidimensional model was compared with the postulated two-factor (structural vs cognitive) and three-factor model (bonding, bridging, linking) in Confirmatory Factor Analyses. The association with self-rated health (0–100 Visual Analogue Scale) and mental distress (GHQ-12 ≥ 4) was assessed in linear and logistic regression models.

**Results:**

A bonding (Cronbach’s a = 0.76), bridging (a = 0.78) and linking (a = 0.89) structure explained 77.6% of the variance and was a better fit as indicated by goodness of fit indices. Elevated odds of mental distress and poorer self-rated health were observed among participants with the lowest levels of perceived workplace social capital. In adjusted models, associations appeared stronger with bonding social capital (adjOR of mental distress = 2.71 95% CI = 1.08, 6.79) while those with the highest scores rated their health higher by 8.0 points on average (95% CI = 2.1, 13.8). Low linking social capital was also associated with poorer health but no consistent associations were observed with bridging.

**Conclusion:**

While associations appeared stronger with bonding and linking, this may reflect a weakness of the measure to fully capture bridging social capital. Even though, this aspect might need strengthening, the WSC showed good metric properties in a different setting, language and occupational group. Cross-national and cognitive validation studies are needed.

## Background

There has been an ongoing debate with regards to the theoretical definition of social capital, and hence its operational measurement [1]. Murayama et al. [2] pointed out that all definitions share common features in that they mainly refer to two aspects. Firstly, the social structure of a community or similarly a workplace, a school, or any other social grouping (i.e. the density and other structural features of social networks that promote social ties) and, secondly, to the norms and attitudes of the group. Among these, trust and reciprocity lie at the core, as they are thought to provide resources and opportunities by virtue of membership. One of the most cited definitions is *features of social organization that facilitate coordination and cooperation for mutual benefit* [3].

While social capital has been extensively studied in the community [4, 5], in recent years, there has been a natural interest in extending the study of social capital as an important aspect of a healthy work environment. Related, but more restrictive concepts, have been long considered essential features in well-accepted theoretical models of work environment, e.g. social support [6]. To date, studies in several work settings have reported associations between low levels of social capital, either measured at the level of the individual (i.e. perception) and/or aggregately at the level of the unit (i.e. a contextual characteristic) with low self-rated health [7, 8], depressive symptomatology [9], hypertension [10], health-threatening behaviours [8, 11] and long-term sickness absence [12].

However, there is no uniformity in terms of measurement. In addition, studies originate from a small variety of settings, precluding an assessment whether findings are culturally-specific. A 2013 review identified 17 studies in general settings, of which 11 originate from Finland (public sector employees across different occupational groups) and 3 from Japan (commonly private company employees, while the specific sector is not always named) [13]. An additional 7 studies, generally though with smaller sample sizes, were set in hospitals or other health care facilities (such as nursing homes), targeted clinicians and nurses, 5 of which from Germany [13].

Some of these studies have used short multi-item scales (6–8 items), which nevertheless tend to focus on certain cognitive features of social capital at work, such as support, trust and reciprocity [14]. A number of studies seem to use single-items to operationalise constructs such as Trust:: “I trust the people I work with” or “would you say that most people in your company can be trusted or that you can’t be too careful in dealing with people?”, Reciprocity: “The people I work with are willing to help each other” or “Would you say that most of the time people in your company try to be helpful, or that they are mostly just looking out for themselves?” [8, 11]. A number of studies even use specific workplace characteristics as postulated proxies of social capital, such as employment security [15].

The Finnish Public Sector Study (FPSS) is by far the largest with participants from several sectors of public services. While according to the authors as many as 1800 different occupational titles were included in the series of studies, registered and practical nurses in state hospitals were by far the biggest occupational group, representing 36% of the total sample, followed by teachers at 19%. In these studies, a psychometrically tested scale was used to assess workplace social capital [16].

Albeit also short, the FPSS Workplace Social Capital (WSC) scale seems to have a good face validity. Unlike other scales which tend to focus on specific cognitive aspects of social capital (such as common values and perceived trust), or related constructs (such as justice), the FPSS WSC is based on an integrated theoretical model of social capital and hypothesized to tap on both structural i.e. what people do and cognitive aspects of social capital i.e. how people feel [17]. Moreover, it incorporates items that are thought to tap on all types of social capital, namely bonding, defined as relations within a group of similar social identity, bridging defined as co-operative relations and mutuality across such groups who may be dissimilar in social characteristics, and linking, defined as connections across different power or status hierarchies [3].

The FPSS Workplace Social Capital (WSC) scale was recently translated and used in Japan [18] and China [19], showing cross-sectional associations with obesity and mental health respectively. In the latter of these studies, the WSC was used as a unidimensional construct, while in the other the postulated sub-dimensions of bonding, bridging and linking were used, without testing the dimensionality of the scale.

### Aim

This study explored the cross-cultural transferability of the FPSS Workplace Social Capital (WSC) questionnaire in a different setting (Cyprus), language (Greek) and occupational group (nurses). Specifically, the study investigated the construct validity of the scale based on the postulated two- and three-factor model, the internal consistency of the scale’s items as well as its criterion validity according to the observed cross-sectional association of individual perception of workplace social capital with self-rated health and psychological distress.

## Methods

### Study design, participants and sampling

The target population was Registered Nurses (RN) who attended an upgrade degree programme offered one-off to all diploma graduates. With the establishment of the Department of Nursing at the Cyprus University of Technology, there was a political decision to seize the award of the three-year diploma in Nursing (offered by the old Nursing School). Whilst not compulsory, due to the Unions’ interest in preserving equal opportunities, in view of the first University graduates, the overwhelming majority of the registered nursing workforce on the island from the public and private sector attended this nationwide programme with over 3500 graduates. This offered a natural opportunity to access the target population with limited resources. Initial calculations indicated that a sample of 300–360 (10% of the target population) was sufficient to provide 90% power to detect a statistically significant correlation of as little as 0.15 between social capital and the study outcomes as well a difference in self-rated health of 0.3 SD and an odds ratio of mental distress in the magnitude of 1.5 between low and high levels of social capital. Furthermore, it was adequate in order to test the metric properties of this 8-item scale (more than 30 participants per item).

Nationwide, there were 28 class groups of varying sizes (80–200), reflecting the size of the working force in each district. The groups attended a series of eight courses with no preset order; thus attending a specific course during a specific semester was largely random. A two-stage sampling procedure was used by which a random sample of class groups (*N* = 8) stratified by district (*N* = 4) was first selected. Specifically, four out of 10 groups were randomly selected from the largest district, two out of six groups from the second largest district, and one out of three groups from the two smallest districts. Based on class lists, the total number of registered participants in these 8 class groups was 775, with numbers per district proportional to the actual distribution of the total working force (i.e. approximately 50%, 30%, 10% and 10%). The sample size was purposefully inflated in order to account for the expected low class attendance. In the second-stage, all nurses who attended the class the day of the survey were eligible to participate.

### Measurement tools

Participants responded to the WSC scale which consists of eight 5-point Likert scale items. The scale was forward translated into Greek from English (not Finnish) independently by two of the authors and back-translated independently by other two authors. In addition, participants: 1) completed the General Health Questionnaire (GHQ-12), a widely-used non-specific measure of mental distress and 2) self-rated their current state of health on a 0–100 Visual Analogue Scale (VAS). Participants also provided information about their age, gender, marital status, number of children, monthly family income, length of employment, as well as residential status (i.e. tenure and mobility in the last 10 years), as an additional indicator of social position since the sample was homogeneous in terms of educational attainment.

### Data collection and ethical considerations

An information leaflet explaining the purpose of the study was handed out and participation was anonymous and voluntary. No personal identifiers were included, including place of work, position or rank. While this precludes from investigating unit-level (i.e. hospital and/or ward) social capital, it was deemed necessary. Due to the relatively small workforce, such information would be perceived as personal identifiers and might have adversely affected the response rate. Participants completed the questionnaire during the scheduled break and returned it sealed in an envelope at the end of class. Permission to use the questionnaires was obtained through personal communication with the Finnish team (A. Kouvonen) for the FPSS Workplace Social Capital and by GI Assessments for GHQ-12.

### Statistical analysis

Factor validity was assessed in Confirmatory Factor Analysis (CFA). The fit of a unidimensional (overall scale), two-factor (i.e. structural vs cognitive SC) and three-factor (i.e. bonding, bridging, linking) models were compared based on goodness of fit indices (GFI, NFI, CFI and RMSEA). Exploratory Factor Analyses (EFA) were also performed in order to further assess the dimensionality of the scale. The criteria for the number of factors to be retained were: eigenvalues greater than 1, examination of the scree plot and factor loadings of 0.4 or higher. The internal consistency of the scale and subscales were assessed using Cronbach’s alpha coefficient. Item-item and item-total correlations were also examined.

Consistent with previous studies, mean score was calculated across the 8 items as well as per identified factor. Differences in mean WSC as well as mean self-rated health and proportion with high psychological distress (defined as GHQ-12 ≥ 4) by socio-demographic characteristics were assessed in one-way ANOVA and chi-square tests as appropriate, calculating *p*-value for trend across ordinal categories where necessary. Participants with missing values on any of the items of the WSC were excluded from the factor analyses, while regression models were based on the number of participants with no missing values on all variables.

In the absence of cut-off values and in agreement with previous studies [18, 19], the tertile distribution of overall WSC scores corresponding to < 3.5, 3.5–4 and > 4 was used for regression analyses. For consistency, the same values were used in models of sub-scales of WSC, even if they did not exactly correspond to tertile values. The odds of mental distress (defined as GHQ-12 ≥ 4) across decreasing levels of perceived WSC were estimated in logistic regressions models before and after adjusting for socio-demographic variables. The tertile of highest scores was used as reference to express the association as increased odds of distress. Multivariable models adjusted for age, gender, income, marital status, number of children, length of employment, house and type of tenure. Of the two variables for residential instability, length of residence in current address and change of address in last 10 years, only the latter was included in the models due to collinearity. Similarly, the mean difference in self-rated health across increasing levels of social capital was estimated in linear regression models before and after adjusting for socio-demographic variables, using the tertile of highest scores as reference. Models were repeated with categories of WSC as an ordinal variable, as well as with WSC scores as continuous variables in order to express the associations in terms of a 1 SD increase.

## Results

### Descriptive results

A total of 362 questionnaires were returned. This represents 48% of the registered class list of the selected groups (range 24–67% across class groups). Nevertheless, as expected, non-participation was almost exclusively due to non-attendance on the day the questionnaires were distributed. Based on signed class attendance sheets, the response rate among those present on the day the questionnaires were distributed was over 90% across all districts. Missing values on the WSC items, which appeared at the top of the pack, were low (around 1–2%). A higher percentage of participants did not respond to some of the socio-demographic questions (6–10%), the GHQ-12 and self-rated health scale (13–14%). Three in four participants were women (which corresponds to the expected gender distribution) and 72% were married or cohabiting – see Table 1. As many as 30% were relatively new in the work force, while income varied substantially with 12.7% reporting a family income lower than 2000 euros monthly and 10.5% reporting an income higher than 5000 euros. As many as 84.5% of participants were home-owners and 39.5% had been living in the same address for more than a decade.

**Table 1:** Workplace Social Capital mean (SD) by participant characteristics (*N* = 362)

		N	%	WSC Mean (SD)	p (p trend)^†^
Gender	Male	64	17.7%	3.69 (0.70)	
	Female	273	75.4%	3.56 (0.68)	
	Not specified	25	6.9%	3.38 (0.71)	0.17
Age	< 35	128	25.4%	3.45 (0.75)	
	35–45	86	23.8%	3.65 (0.59)	
	45+	121	33.4%	3.64 (0.69)	
	Not specified	27	7.5%	3.54 (0.67)	0.08 (0.02)
Marital status	Single	41	11.3%	3.63 (0.81)	
	Married/Cohabiting	261	72.1%	3.57 (0.68)	
	Divorced/Widowed	26	7.2%	3.62 (0.58)	
	Not specified	34	9.4%	3.43 (0.71)	0.67
Number of children	None	58	16.3%	3.46 (0.83)	
	One	51	14.1%	3.58 (0.64)	
	Two	124	34.3%	3.66 (0.63)	
	Three or more	102	28.5%	3.54 (0.70)	
	Not specified	25	9.9%	3.47 (0.64)	0.41 (0.49)
Family monthly income	< 2000 euro	46	12.7%	3.54 (0.70)	
	2001–3000 euro	113	31.2%	3.50 (0.70)	
	3001–5000 euro	135	37.3%	3.63 (0.66)	
	> 5000 euro	38	10.5%	3.60 (0.72)	
	Not specified	30	8.3%	3.57 (0.73)	0.68 (0.28)
Length of employment	< 3 years	109	30.1%	3.49 (0.70)	
	3–10 years	119	32.9%	3.54 (0.70)	
	More than 10 years	110	30.4%	3.69 (0.67)	
	Not specified	24	6.6%	3.46 (0.68)	0.14 (0.04)
Length of residence in current address	< 3 years	53	14.6%	3.49 (0.75)	
	3–10 years	143	39.5%	3.48 (0.66)	
	> 10 years	143	39.5%	3.70 (0.68)	
	Not specified	23	6.4%	3.51 (0.69)	0.04 (0.01)
Change of address in last 10 years	Never	143	39.5%	3.70 (0.68)	
	Once	117	32.3%	3.50 (0.65)	
	At least twice	74	20.4%	3.43 (0.76)	
	Not specified	28	7.7%	3.53 (0.64)	0.03 (0.004)
House tenure	Owner-occupied	306	84.5%	3.58 (0.69)	
	Privately renting	26	7.2%	3.50 (0.77)	
	Not specified	30	8.3%	3.51 (0.68)	0.76
Type of accommodation	Detached House	224	61.9%	3.55 (0.69)	
	Small apartment block	100	27.6%	3.64 (0.67)	
	Large apartment block	14	3.9%	3.42 (0.82)	
	Not specified	24	6.6%	3.53 (0.69)	0.60
Self-rated health (VAS 0–100)	< 69	108	29.8%	3.45 (0.64)	
	70–79	75	20.7%	3.53 (0.62)	
	80–89	88	24.3%	3.59 (0.74)	
	> 90	41	11.3%	3.85 (0.69)	
	Not specified	50	13.8%	3.62 (0.75)	0.03 (0.003)
Psychological distress (GHQ-12)	< 4	224	61.9%	3.62 (0.65)	
	4 or more	92	25.4%	3.38 (0.74)	
	Not specified	46	12.7%	3.69 (0.73)	0.01

^†^*p*-value of chi-square test (including “not specified”) and, where appropriate, *p*-value for trend across ordinal levels (excluding “not specified”)

### Workplace social capital and association with socio-demographic variables

Perceived social capital in the workplace (WSC) appeared moderate, averaging around 3.5 (SD 0.69) on a theoretical range of 1–5. Older participants had higher scores, and there was a stepwise increase in terms of length of employment (see Table 1). No differences were observed in terms of marital status, number of children, or income. Even though the scale refers to the work setting, a statistically significant stepwise increase was observed in terms of residential stability.

One in three participants self-rated their health below 70 on a 0–100 VAS scale and, similarly, as many as 25.4% had a score of 4 or higher on the GHQ-12. There appeared to be a stepwise increase in perceived WSC from M = 3.45 (SD 0.64) to M = 3.85 (SD 0.69) among those with the lowest (< 69) and highest (> 90) VAS scores respectively (*p*-value for trend = 0.003). Similarly, those with higher levels of psychological distress (4 or more on the GHQ-12) reported on average lower levels of perceived social capital at work (M = 3.38 Vs M = 3.62; *p* = 0.01).

Female participants rated WSC somewhat lower than males, but the observed difference was not statistically significant. Female participants also rated their health somewhat lower (M = 75.9 SD = 18.6) than males (M = 81.3 SD = 12.6; *p* = 0.01). Similarly, levels of mental distress appeared elevated among female participants – 31.0% with GHQ-12 ≥ 4 compared to 19.7% in males; *p* = 0.08. Other than income, for which a social gradient was observed in terms of mental distress (43.2%, 33.3%, 21.4% and 23.5%; *p* = 0.03 across income categories), no statistically significant differences were observed in self-rated health or distress according to other socio-demographic characteristics – not shown in detail.

### Individual items and sub-dimensions of WSC scale

Table 2 presents the relative frequencies of responses for each item of the scale as well as summary statistics in terms of a 2-factor and 3-factor model. No ceiling or floor effects were observed. In general, lower levels of bridging social capital (M = 3.47 SD 0.77) where observed compared to bonding (M = 3.66 SD = 0.71) and linking social capital (M = 3.55 SD = 0.94). Correlations between individual items of the scale ranged between 0.35 and 0.80. In general, items correlated higher with the other items in the same dimension rather than across dimensions, at least based on the 3-factor model (see Table 3). All items correlated with the overall score (0.62–0.80). Cronbach’s α coefficient of internal consistency was estimated at 0.89 for the overall scale, and was comparable to the original study, as well as α = 0.94 in Gao et al. [19] and α = 0.90 in Kobayashi et al. [18]. Deleting any item from the scale did not result in higher values. The figures were 0.76, 0.78 and 0.89 for bonding, bridging and linking SC, also comparable to Kobayashi et al. [18].

**Table 2:** Summary statistics for Workplace Social Capital items, overall scale and sub-scales in either a two-factor (i.e. Structural, Cognitive) or three-factor (i.e. Bonding, Bridging, Linking) models

	2 or 3 factor model^c^		Mean (SD)	Relative frequency (%) of responses^a,b^				
	2	3		1	2	3	4	5
Item 1 – Our supervisor treats us with kindness and consideration.	S	L	3.56 (1.05)	4.1%	13.3%	21.0%	43.7%	16.6%
Item 2 – Our supervisor shows concern for our rights as an employee.	S	L	3.57 (1.03)	3.0%	13.3%	23.8%	40.6%	17.4%
Item 3 – We have a ‘we are together’ attitude.	C	B	3.56 (0.98)	3.3%	13.0%	19.3%	51.1%	12.2%
Item 4 – People keep each other informed about work-related issues in the work unit.	S	B	3.96 (0.74)	0.6%	5.0%	11.3%	63.3%	18.5%
Item 5 – People feel understood and accepted by each other.	C	B	3.45 (0.87)	0.6%	15.8%	27.6%	47.0%	7.2%
Item 6 – Do members of the work unit build on each other’s ideas in order to achieve the best possible outcome?	S	Br	3.49 (0.85)	0.3%	14.4%	27.6%	47.8%	7.5%
Item 7 – People in the work unit cooperate in order to help develop and apply new ideas.	S	Br	3.43 (0.86)	1.7%	12.7%	31.2%	45.9%	6.6%
Item 8 – We can trust our supervisor.	C	L	3.51 (1.03)	3.6%	12.7%	27.6%	38.1%	16.0%
			Mean	SD	Med	Min	IQR	Max
Overall scale			3.57	0.69	3.63	1.6	3.0–4.0	5
Structural SC			3.53	0.70	3.60	1.8	3.2–4.0	5
Cognitive SC			3.51	0.80	3.67	1.0	3.0–4.0	5
Bonding SC			3.66	0.71	3.83	1.3	3.3–4.0	5
Bridging SC			3.47	0.77	3.50	1.0	3.0–4.0	5
Linking SC			3.55	0.94	3.67	1.0	3.0–4.0	5

Notes – ^a^1 = fully disagree to 5 = fully agree; except item 7 where 1 = very little to 5 = very much, ^b^Percentages do not add up to 100% due to missing values. Missing values were very low and ranged from 4 (1.1%) to 7 (1.9%), ^c^Items hypothesized to capture S = structural and C=Cognitive Social Capital in a 2-factor model or B=Bonding, Br = Bridging and L = Linking Social Capital in a 3-factor model

**Table 3:** Item-item correlation, item-total correlations and Cronbach’s alpha coefficients if item deleted for the eight items of the Workplace Social Capital scale

	2 or 3 factor model		Item1	Item2	Item3	Item4	Item5	Item6	Item7	Item8	Item-Total	Alpha, if item deleted
Item1	S	L	1.0								0.78	0.87
Item2	S	L	*0.80*	1.0							0.81	0.83
Item3	C	B	0.40	0.44	1.0						0.75	0.87
Item4	S	B	0.34	0.42	0.43	1.0					0.62	0.88
Item5	C	B	0.40	0.43	*0.66*	0.45	1.0				0.73	0.87
Item6	S	Br	0.40	0.43	0.53	0.39	0.50	1.0			0.71	0.88
Item7	S	Br	0.50	0.48	0.53	0.42	0.44	*0.64*	1.0		0.74	0.87
Item8	C	L	*0.69*	*0.72*	0.50	0.41	0.52	0.39	0.47	1.0	0.80	0.86
			Cronbach’s Alpha				Cronbach’s alpha				Cronbach’s alpha	
Overall SC (all items)			0.89	Structural SC (items 1,2,4,6,7)			0.82	Bonding SC (items 3,4,5)			0.76	
				Cognitive SC (items 3,5,8)			0.79	Bridging SC (items 6,7)			0.78	
								Linking SC (items 1,2,8)			0.89	

Note: *p*-values for all Pearson correlations < 0.001. Item-item correlations higher than 0.60 appear in the Table in italic

### Dimensionality of the WSC scale

Kaiser–Meyer–Olkin coefficient for sampling adequacy was 0.855 and the Barlett’s test of sphericity was statistically significant (*p*-value < 0.001), supporting that the data are appropriate for factor analysis. In CFA, a three-factor solution (i.e. bonding, bridging, linking) appeared to be a better fit as indicated by all goodness of fit indices (GFI, NFI, CFI and RMSEA) compared to both a unidimensional model and a two-factor solution (i.e. structural vs cognitive SC). Even though all indices appeared substantially improved for the three-factor model, some of indices did not suggest a good fit in absolute terms (e.g. Root Mean Square Error of Approximation = 0.091).

In Exploratory Factor Analysis, a clear structure was observed after orthogonal rotation. Two factors were initially extracted with an eigenvalue greater than one, explaining 68.9% of the variance. The three items corresponding to linking SC (i.e. referring to the ‘supervisor’) loaded on the first factor, while all the rest loaded on the second factor. After examination of the scree plot, three factors were extracted and rotated resulting in the postulated structure of bonding (explaining 25.0% of the variance), bridging (22.2%) and linking (30.4%), explaining 77.6% of the variance. Other than the item referring to having “a together attitude”, which loaded on both bonding and bridging, no cross-loading was observed. While conceptually this is understandable, there was a large discontinuity between the loading of this item on bridging (0.44) compared to the next higher (0.80). Table 4 presents the findings with regard to exploratory and confirmatory factor analysis.

**Table 4:** Rotated component matrix for exploratory factor analysis of the Workplace Social Capital questionnaire and confirmatory factor analysis fit measures for a unidimensional, two- and three-factor models

A. Exploratory Factor Analysis								Factor 1 - Linking		Factor 2 - Bonding		Factor 3 – Bridging
Item 1 – Our supervisor treats us with kindness and consideration.						S‡	L‡	0.89				
Item 2 – Our supervisor shows concern for our rights as an employee.						S	L	0.88				
Item 8 – We can trust our supervisor.						C	L	0.78				
Item 5 – People feel understood and accepted by each other.						C	B			0.79		
Item 4 – People keep each other informed about work-related issues …						S	B			0.74		
Item 3 – We have a ‘we are together’ attitude.						C	B			0.69		0.44
Item 6 – Members … build on each other’s ideas … best possible outcome						S	Br					0.84
Item 7† – People … cooperate in order … develop & apply new ideas						S	Br					0.80
Initial eigenvalues								4.45		1.06		0.70
Total variance explained (77.6%)								30.4%		25.0%		22.2%
B. Confirmatory Factor Analysis								CMIN/DF	GFI	NFI	CFI	RMSEA (90% CL)
Unidimensional								16,689	0.767	0.779	0.789	0.213 (0.193, 0.233)
Two factor model	Structural		Cognitive					16,619	0.763	0.791	0.800	0.212 (0.192, 0.233)
Three factor model	Bonding	Bridging		Linking				3881	0.956	0.956	0.967	0.091 (0.069, 0.115)

^†^All 5-point Likert scale labeled 1 = fully disagree to 5 = fully agree; except item 7 where 1 = very little to 5 = very much

^‡^Items hypothesized to capture S = structural and C=Cognitive Social Capital in a 2-factor model or B=Bonding, Br = Bridging and L = Linking Social Capital in a 3-factor model

### Association of WSC with self-rated health and psychological distress

Higher odds of distress and lower self-rated health were consistently observed among participants with the lowest levels of perceived social capital at work – see Table 5. The tertile of participants with the lowest WSC scores were 2.16 times (95% 1.05, 4.42) more likely to score 4 or higher on the GHQ scale while on average they rated their health lower by 8.4 points (95% CI 2.8, 14) on a 0–100 VAS. After adjusting for age, gender, income, marital status, number of children, length of employment, residential instability, house and type of tenure, association with self-rated health slightly attenuated, but remained statistically significant. In fact, there appeared to be a stepwise increase in self-rated health of 3.2 (95% 1.1, 5.3) per 1 SD increase in WSC (*p* < 0.01). In contrast, elevated odds of psychological distress appeared restricted to those with the lowest levels of WSC. While the odds ratio estimate only slightly attenuated to 1.95 (95% CI 0.88, 4.25), it was no longer statistically significant in the adjusted model (see Table 5*).*

**Table 5:** Association of workplace social capital and self-rated health and psychological distress (GHQ-12)

	Self-rated health - VAS 0–100 (*N* = 280)			Psychological distress – GHQ-12 ≥ 4 (*N* = 283)	
	Unadjusted Mean	Adjusted Mean^†^	Mean Difference (95% CI)	Unadjusted OR (95% CI)	Adjusted OR^‡^ (95% CI)
WSC – Overall scale					
Lowest (< 3.5)	73.9	75.3	−8.4 (−14.0, −2.8)	2.16 (1.05, 4.42)	1.93 (0.88, 4.25)
Middle (3.5–4.0)	77.0	78.1	−5.6 (−10.9, −0.2)	1.03 (0.49, 2.12)	1.04 (0.47, 2.32)
Highest (> 4.0)	82.6	83.6	Ref	Ref	Ref
Per tertile increase (95% CI)	4.2 (1.5, 6.9), *p* < 0.01	4.1 (1.3, 6.8), *p* < 0.01		0.64 (0.45, 0.92)	0.68 (0.46, 1.01)
Per 1 SD increase (95% CI)	3.0 (1.0, 5.1), *p* < 0.01	3.2 (1.1, 5.3), *p* < 0.01		0.72 (0.55, 0.93)	0.76 (0.57, 1.01)
Bonding SC					
Lowest (< 3.5)	74.6	75.4	−8.0 (−13.8, −2.1)	2.94 (1.26, 6.89)	2.71 (1.08, 6.79)
Middle (3.5–4.0)	76.9	77.6	−5.7 (−11.3, −0.0)	2.39 (1.03, 5.58)	2.69 (1.07, 6.77)
Highest (> 4.0)	82.6	83.3	Ref	Ref	Ref
Per category increase (95% CI)	3.7 (0.9, 6.6), *p* = 0.01	3.7 (0.9, 6.6), *p* = 0.01		0.64 (0.44, 0.93)	0.69 (0.47, 1.03)
Per 1 SD increase (95% CI)	2.9 (0.9, 5.0), *p* < 0.01	2.8 (0.8, 4.9), *p* = 0.01		0.78 (0.60, 1.00)	0.83 (0.63, 1.10)
Bridging SC					
Lowest (< 3.5)	76.2	76.9	−2.1 (−9.2, 5.0)	0.97 (0.41, 2.29)	0.87 (0.34, 2.24)
Middle (3.5–4.0)	78.3	79.5	0.5 (−6.9, 7.9)	0.71 (0.29, 1.78)	0.64 (0.23, 1.77)
Highest (> 4.0)	78.2	79.0	Ref	Ref	Ref
Per category increase (95% CI)	1.4 (−1.7, 4.5), *p* = 0.39	1.6 (−1.5, 4.7), *p* = 0.31		0.91 (0.62, 1.34)	0.94 (0.62, 1.43)
Per 1 SD increase (95% CI)	1.4 (−0.7, 3.5), *p* = 0.20	1.6 (−0.5, 3.8), *p* = 0.13		0.77 (0.60, 1.00)	0.82 (0.62,1.08)
Linking SC					
Lowest (< 3.5)	74.0	75.0	−9.8 (−15.1, −4.6)	2.07 (1.05, 4.11)	1.90 (0.89, 4.07)
Middle (3.5–4.0)	76.3	77.1	−7.7 (−13.2, −2.2)	0.97 (0.46, 2.04)	1.05 (0.56, 2.43)
Highest (> 4.0)	84.2	84.8	Ref	Ref	Ref
Per category increase (95% CI)	4.8 (2.3, 7.4), *p* < 0.01	4.6 (2.0, 7.2), *p* < 0.01		0.65 (0.56, 0.92)	0.70 (0.48, 1.01)
Per SD increase (95% CI)	2.9 (0.8, 5.0), *p* < 0.01	3.1 (0.9, 5.2), *p* < 0.01		0.73 (0.56, 0.95)	0.75 (0.56, 1.00)

^†‡^Adjusted for age, gender, income, marital status, number of children, length of employment, change of address in last ten years, house and type of tenure. ^†^Increasing age, female gender and lower income were statistically significantly associated with lower self-rated health in multivariable models. ^‡^Female gender, lower income and not owner occupants had statistically significantly higher odds of GHQ-12 ≥ 4 in multivariable models

Similar, if not stronger, associations were observed with bonding social capital. The adjusted OR of psychological distress was 2.71 (95% CI 1.08, 6.79) among those with the lowest scores, while participants with the highest scores rated their self-rated health higher by 8.0 points on average (95% CI = 2.1, 13.8). Low linking social capital also appeared to be significantly associated with poorer general and mental health, but no consistent associations were observed with bridging social capital.

## Discussion

### Main findings

The 8-item FPSS WSC questionnaire showed good metric properties (construct validity, internal consistency and criterion-validity in terms of concurrent association with self-rated health and psychological distress). The observed dimensionality appeared more consistent with a bonding, bridging and linking typology, rather than a structural-cognitive typology. Even though associations with poorer self-rated health and higher levels of distress appeared stronger with perceived bonding and linking, rather than bridging social capital, this might also reflect a range of measurement issues, or its use in the specific occupational group (registered nurses) or setting (hospitals).

### Strengths and limitations

The study design precludes any inference on causal associations. Nevertheless, the purpose was to test the cross-cultural transferability and postulated dimensionality of the scale in a new language, setting and occupational group. A limitation is the fact that the study targeted only one occupational group (registered nurses). Nevertheless, it was expected that the nationwide coverage of several work settings would introduce enough variability for the purposes of assessing the dimensionality of the scale.

It should be noted that information on place of employment was not collected in order to preserve anonymity and encourage participation. As a result, only the individuals’ perception of workplace social capital was assessed. Exploring the aggregate workplace social capital of specific hospitals and units was not the focus of the current study. The sampling frame in this study was based on class groups, comprising of nurses from several settings (hospitals, clinics, rehabilitation units, community health care centres, mental health services etc), operating in the public and private sector. One of the most enduring debates in the social capital literature is whether social capital is the property and resource for the individual, or whether it is should be conceptualized as a contextual characteristic, and thus measured at the group (community, work unit) level, or both [20]. Future studies should use sampling frames that allow the measurement of workplace social capital at the individual as well as the unit level. Interestingly, previous multilevel studies incorporating both an individual’s perception as well as group-level social capital (expressed as the aggregate responses of the members of the work unit) commonly report stronger associations with individual, rather than contextual, WSC [8, 9, 19].

Furthermore, reverse causation cannot be excluded i.e. people may have a different experience of their work environment or at least perceive it as less cohesive as a result of psychological distress or poorer health. Also, while the response rate was high among class attendees, it is likely that people who systematically attended classes are a select sub-group. For instance, people who skipped classes might be those who could depend on colleagues to share the lecture notes. Even though, selection bias is possible, the intention was neither to provide an estimate for either perceived levels of social capital or prevalence of poor health or distress. Nevertheless, this self-selection bias might have also impacted the results due to restriction in the range of WSC scores. Even though, it seems that there was no floor or ceiling effects, and the participants’ responses were distributed across all the full range of responses for all eight items, in the absence of any other studies of workplace social capital in this, or any other occupational groups for this matter in Cyprus, the extent to which this represents the expected variability in WSC is not known.

### Association of social capital in the workplace with the health of employees

This study set out to explore the metric properties of the Workplace Social Capital scale in a different socio-cultural setting among a well-defined occupational group. The FPSS WSC scale had so far been used only in select linguistic and socio-cultural contexts (Finland, Japan and China). Other than the construct validity of the scale, the study investigated the criterion validity according to the observed association with self-rated health and psychological distress, as measured by the GHQ scale. Previous studies of workplace social capital explored a wide range of different health outcomes. In the original Finnish Public Sector Study, associations were observed between low baseline levels of workplace social capital or a decline in social capital during follow-up with depression [9], self-rated health [7] and likelihood to quit smoking [21], but not the co-occurrence of lifestyle risk factors e.g. smoking, drinking, overweight and physical activity [22]. Studies from the same group also linked low workplace social capital with risk of hypertension at least among men [10] and even increased risk of all-cause mortality [23].

Beyond the FPSS, we are aware of only two studies that have used the same tool. Consistently with the findings of this study, in the cross-sectional study by Gao et al. [19] a more than three-fold increase in the odds of poor mental health was reported among Chinese employees with the lowest levels of social capital. It should be noted, however, that the results of this study cannot be directly compared to previous studies since either different occupational groups were studied and/or different scales to measure workplace social capital were used and/or different health outcomes were considered or different measurement tools were used for the same study outcome. For instance, while in the current study, self-rated health was measured using a 0–100 VAS, previous studies commonly assessed self-rated health on a 5-point response scale from Poor to Excellent, which was then commonly dichotomised to “more” or “less than good” [7, 8]. Similarly, even though the study by Gao et al. [8], used the same WSC scale, it measured mental well-being using the WHO-Five Well-Being Index [19].

The GHQ, used in this study, is a commonly used screening tool for depressive symptomatology. There is plenty of evidence to suggest that perceptions of psychosocial stressors in the workplace are related to an elevated risk of subsequent depression or depressive symptomatology [24]. Traditionally studies in occupational settings examine job strain and effort-reward imbalance on mental health; however, they are not commonly set in a social capital framework. A recent exception is the prospective study of 8000 employees across 12 companies in Japan (the J-Hope study), which provided evidence to suggest that an increase in workplace social capital, measured using a 6-item scale of cognitive social capital was associated with an improvement in psychological distress, measured by the Kessler Psychological Distress scale, even after adjusting for work environment characteristics [25, 26]. Even though the study used a different scale to measure workplace social capital, it is important to note that most of the questions resemble closely those of the FPSS WSC questionnaire used here.

It is currently not clear whether all aspects of social capital (e.g. structural vs cognitive, or bonding, bridging, linking) carry the same weight [27]. In this study we explored the purported dimensionality of a workplace social capital scale and compared a unidimensional scale with a two dimension (structural-cognitive) and a three dimension model (bonding, bridging, and linking), which appeared to be a better fit. With some exceptions [18, 28], most published studies use an overall score to represent workplace social capital, as in Tsuboya et al. [25], rather than investigate these dimensions separately. The cross-sectional survey of Kobayashi et al. [18] is one of these exception. Using the same scale as in this study, it showed beneficial effects of overall, bonding and linking WSC on the prevalence of overweight, at least among male employees. Interestingly, like in the case of this study, no association was observed with bridging social capital. The authors attributed this lack of association to the limited heterogeneity in social class differentials among the study sample. While the specific sector in that study is not named, it is likely that in the particular sector cooperation across different occupational (and social) groups is not as an integral part of the organization’s function as it is among an inter-disciplinary health team, often with power differentials.

Since the aim of the study was to assess the metric properties of the WSC for potential use in future studies, we did not explicitly explore the underlying mechanisms by which workplace social capital may affect health. It is generally hypothesized that WSC may serve as a moderator of job stresses and other adverse psychosocial work conditions on health [11]. At the same time, it is also likely that WSC may act as a mediator. A study among 9350 Japanese employees found evidence of both processes [29]. Individual perceptions of WSC mediated the association between adverse work characteristics (mainly with regards to lack of supportive resources) and psychological distress. At the same time, there was evidence of a moderation effect of social capital on the association between external pressures (such as high job demands, strain, effort, and effort-reward imbalance) and psychological distress, but only when levels of social capital were highest.

### Workplace social capital among health sector employees

It is only natural that a number of studies have focused specifically on the healthcare sector since the literature on job satisfaction, burn-out, retention of staff, and quality of care from clinical settings is vast, and has long recognized the importance of contextual factors, such as organizational dimensions of the work environment, without however always directly addressing the social dynamics involved [30].

Unlike studies in the general working force, studies from clinical settings tend to be smaller and, like the current study, cross-sectional in design. These studies have provided evidence to suggest that aspects of the social environment in health organization are associated with the well-being of the staff as well as the performance of the organization. Nevertheless, comparison of findings across studies in clinical settings is limited as studies do not follow a common unified framework. In fact, they tend to measure different and select aspects of social capital, some considered to be antecedents, such as communication and trust, and their association to a wide range of outcomes. For example, a series of cross-sectional studies from Germany has provided evidence that low “social capital” among clinicians and nurses, defined here as “common values” and “perceived trust”, was associated with emotional exhaustion [31, 32], lower job satisfaction [33] and lower clinical risk management [34]. Similarly, a study among 239 workers in 11 nursing homes in Belgium measured social capital by six items referring to vertical trust, justice and social community at work (drawn from the Copenhagen Psychosocial Questionnaire) [35]. Spence Laschinger and Read [36] developed a theoretical model where structural empowerment (defined as access to opportunities, resources, information, support and formal and informal power measured by the Conditions of Work Effectiveness Questionnaire) and social capital at unit-level were associated with unit effectiveness (ability to provide timely care) as well as perceived patient care quality. They measured social capital using 9 items that relate to structural, relational and cognitive aspects of social capital.

### Measurement of workplace social capital

The continuing ambiguity around measurement issues with regards to the concept of social capital is not a unique feature of studies in occupational settings. In fact, one may argue that due to the fact that the literature is still smaller, studies of workplace social capital have been more consistent than the respective literature in community settings. The WSC scale has been more often used as a unidimensional scale, with some exceptions. For example, in Oksanen et al., [27] it was operationalized as horizontal (bonding and bridging combined) and vertical (corresponding to linking), confirmed using factor analysis such as in this study..In Kobayashi et al. [18] the bonding, bridging and linking sub-scales were used, however without testing the dimensionality of the scale. The observed dimensionality of the WSC scale in the present study appears more consistent with a bonding, bridging and linking typology (rather than a structural-cognitive typology)..

Like in Kobayashi et al. [18], the weakest associations in the current study were also observed with bridging social capital. We hypothesize that this may reflect a weakness of the measure to fully capture bridging social capital both in terms of the scale itself (only 2 items), or perhaps the setting or occupational group under investigation. Specifically, the scale may not adequately capture the social interaction with other health professional groups (e.g. physicians). The WSC in its original format refers to “people”, “members”, and “we”. Even though this is understandable, since in the original study various occupational groups were represented, in-group identification is not explicit as the same terms are used for both bonding and bridging social capital. These terms contain an ambiguity, especially since items pertaining to bonding and bridging refer to “people”. These issues need to be explored further, as it might be worth revisiting and strengthening this aspect of the scale, and even customizing the scale to the specific setting it will be used in. In retrospect, it might have been better in the context of this study if items that tap on bridging were instead phrased as “members of the healthcare team of doctors, nurses etc … build on each other’s ideas etc” and “In this hospital (or ward or unit), members of the healthcare team cooperate”.

Approaching this issue from within a nursing management framework, Sheingold et al. [30] compared the 96-item Integrated Questionnaire for the Measurement of Social Capital (SCIQ) developed by the World Bank with eight instruments referring to social dimensions of the work environment, including the Essential of Magnetism (EOM) tool. They noted that several of the SCIQ items map to currently accepted dimensions in existing questionnaires of the nursing work environment (such as nurse-nurse and nurse-physician interaction, effective communication, empowerment, supportive leadership). However, none adequately measures the nature of social relationships. Hence, social relational aspects of the work environment, central to the concept of social capital, such as the sense of belonging and shared identity, are currently not adequately addressed. They called for work environment instruments that could be anchored within a social capital framework.

The potential role of shared identities in protecting health and well-being as well as the potential to cultivate shared identities as a “social cure” has been extensively described by Haslam et al. [37] and Jetten et al. [38]. It is interesting to draw parallels between the workplace social capital approach and the track of research from social and organizational psychology on social identification. Social identity theory postulates that people define their sense of self in terms of group membership, single or multiple, which may be centred on family, friends, community, religion, political affiliations and/or work. In-group identification is internalized, provides a sense of belonging and purpose and shapes many processes beneficial for health, including the provision of social support and accrual of social capital. Haslam et al. (2018) criticize the social capital approach for neglecting the underlying mechanisms of social pathways to health, since in-group identification, the cognitive component of belonging to a group, is often assumed and not measured [39].

Indeed, “sense of belonging” is a central tenet of social capital. It is somewhat surprising that there are not many published concept analyses of social capital even though there has been such an interest in the concept for a number of years now. A concept analysis by Reed [40], as it specifically relates to nurses’ work environment, identified the essential attributes of social capital as “social relationships”, “shared assets” and “shared ways of knowing and being”, while antecedents include several of the concepts that existing questionnaires measure, such as communication, trust, and even positive leadership practices (which arguably pertains to linking social capital). A comparison between the WSC questionnaire and the attributes identified in the aforementioned theoretical study reveals an interesting parallelism. In terms of “social relationships”, these include relationships with other nurses (i.e. what would normally be termed “bonding”), within the entire healthcare team (i.e. perceived as “bridging” social capital) and across different levels of the management hierarchy (“linking”). In terms of “shared assets”, while this does not feature as a distinct dimension in the WSC scale, most of the assets named are one way or another referred to in the scale’s items, i.e. support (“People feel understood and accepted by each other”), cooperation and teamwork (“People … cooperate in order to help develop and apply new ideas”), and information (“… keep each other informed”). Lastly, in terms of “shared ways of knowing and being”, also referred to as “a sense of collective consciousness or social connectedness” [41], one of the items in the WSC taps onto the fundamental concept of sense of belonging (i.e. People have a “we are together” attitude).

However, in-group identification is not explicitly defined in any of the items of the WSC. The items may tap to some extent on perceived shared identity (social identity) but do not explicitly refer to the individual’s relationship to the group (social identification). For example, the WSC scale’s item “People feel understood and accepted by each other” may tap on cognitive aspects of attributes attached to the group as a group, rather than the extent to which the group is a source of positive emotions for the individual. Similarly, “We have a ‘we are together’ attitude” assumes that the responder sees themselves as part of “we” rather than “I identify with..” or “the [group] is an important part of my identity”,more common in social identification scales.

Unidimensional and multidimensional scales have been developed to measure social identification [42]. Unidimensional scales commonly tap on cognitive and affective aspects of identification to measure the relationship of the individual to the group. They often include phrases such as “having a lot in common” and “liking” other members of the group. Multidimensional scales are more complex and tap on self-investment and its components of centrality (i.e. the group being important to one’s sense of self), satisfaction (i.e. group membership is the source of positive emotions) and solidarity (i.e. attachment and commitment) [43]. Among nurses in particular, in-group identification has been previously measured by a 3-item scale, “I identify with nurses”, “I have a lot in common with nurses” and “Being a nurse is an important reflection of who I am”, all of which tap on centrality, rather than the other components [44]. Nevertheless, Postmes et al. (2013) have shown that the construct is sufficiently homogeneous to be measured by a Single-Item Social Identification measure (SISI): “I identify with my [group]”, measured on a 7-point scale [42] .Such measures could supplement workplace social capital studies in the future. It is often cited that there is much need for experimental studies to document the potential effectiveness of interventions which aim to produce, foster or strengthen social capital. Examples of such interventions in the literature include techniques such as team-building training [45] and work-based group physical exercise [46]. However, it is not clear how these interventions were designed, which type of social capital they are targeting and in what way. Thus, more formative and empirical research is needed in order to delineate the determinants of social capital in the workplace and the underlying mechanisms linking it to well-being in order to design potentially effective interventions. Equally importantly, using a unified framework and the availability of valid measures of workplace social capital, supplemented by social identification measures, should be considered a prerequisite.

## Conclusion

Studies in occupational settings have produced evidence to suggest that aspects of workplace social capital may be important for well-being. Nevertheless, the measurement of workplace social capital has not been consistent. Furthermore, the extent to which the concept of social capital in the workplace, or in the community for that matter, is culturally-specific is not clear. The WSC scale may appear in general to be a promising tool for measuring workplace social capital. However, the results of this study may suggest a weakness of the measure to capture bridging social capital. In-group identification is not explicit, especially since the same terms are used for both bonding and bridging social capital. Future studies might have to modify terms depending on the dimension they are postulated to tap on in order to refer to “nurses” (or other group) instead of “we” and perhaps “other health professionals in the work unit” in this case instead of “members of the work unit” in the bridging dimension.

In this study, the target population was registered nurses, and thus the results are not generalizable to other occupational groups or settings. Nevertheless, with the exception of the weakness of the scale in its current form to capture in-group identification, the tool seem to map well to concept analysis of the construct, at least with regards to nurses’ work environment. More studies, and in fact cross-validation studies across different cultural settings, are warranted. In addition, concept analyses and especially cognitive validation studies are essential for either adapting and strengthening existing tools or developing new. If social capital is going to be a useful measure for assessing the health effects of the social-relational environment at work as well as the potential effects of social capital fostering interventions, the research community needs to invest on measurement but more importantly on clarifying the concepts and the underlying mechanisms further. Both qualitative approaches (who are “we” and who are “the people in the work unit”) as well as quantitative approaches are needed to explore these issues further. For instance, future studies may look into the association between the Workplace Social Capital scale and sub-scales with social identification measures and other related scales to explore the convergent and divergent validity of its subscales, such as nurse-to-nurse interaction with bonding and nurse-to-doctor interaction with bridging.

One advantage of adopting a social capital framework in exploring workplace health is that in addition to bonding (more related to in-group identification), it also explicitly recognizes the role of leadership and organizational factors (linking social capital). Even so, it is currently not clear whether linking social capital in occupational settings should be considered an antecedent of other forms of social capital. A study among nurses in Belgium [47] suggested that organizational support and leader-member exchange was associated with performance, but social identification was a strong moderating factor, defined both as professional identification (sense of “we” as nurses) and organizational identification (sense of “we” as the organization).

## Abbreviations

CFA: Confirmatory Factor Analysis
EFA: Exploratory Factor Analyses
FPSS: Finnish Public Sector Study
GHQ: General Health Questionnaire
NCBC: Cypriot National Committee of Bioethics
RN: Registered Nurses
SC: Social Capital
SD: Standard Deviation
VAS: Visual Analogue Scale
WSC: Workplace Social Capital
